# Transcriptional Regulation of Tetrapyrrole Biosynthetic Genes Explains Abscisic Acid-Induced Heme Accumulation in the Unicellular Red Alga *Cyanidioschyzon merolae*

**DOI:** 10.3389/fpls.2016.01300

**Published:** 2016-08-29

**Authors:** Yuki Kobayashi, Kan Tanaka

**Affiliations:** ^1^Laboratory for Chemistry and Life Science, Institute of Innovative Research, Tokyo Institute of Technology, YokohamaJapan; ^2^Core Research for Evolutional Science and Technology, Japan Science and Technology Agency, SaitamaJapan

**Keywords:** abscisic acid, *Cyanidioschyzon merolae*, heme, tetrapyrrole, transcriptional regulation, TSPO

## Abstract

Abscisic acid (ABA), a pivotal phytohormone that is synthesized in response to abiotic stresses and other environmental changes, induces various physiological responses. Heme, in its unbound form, has a positive signaling role in cell-cycle initiation in *Cyanidioschyzon merolae*. ABA induces heme accumulation, but also prevents cell-cycle initiation through the titration of the unbound heme by inducing the heme scavenging protein tryptophan-rich sensory protein-related protein O. In this study, we analyzed the accumulation of tetrapyrrole biosynthetic gene transcripts after the addition of ABA to the medium and found that transcripts of a ferrochelatase and a magnesium-chelatase subunit increased, while other examined transcripts decreased. Under the same conditions, the heme and magnesium-protoporphyrin IX contents increased, while the protoporphyrin IX content decreased. Thus, ABA may regulate the intracellular heme and other tetrapyrrole contents through the transcriptional regulation of biosynthetic genes.

## Introduction

Abscisic acid (ABA) is a phytohormone of land plants that is involved in many aspects of plant physiology. ABA induces stress tolerance under various stressful conditions, such as drought and high salt (Skriver and Mundy, 1990; Tuteja, 2007). ABA induces the growth of roots, as well as dormancy in buds and seeds, and in leaf organs it induces the stomatal closure, which helps the plant to preserve water during droughts. It is widely believed that these responses are mostly mediated by transcriptional activation, and an underlying mechanism involving the specific receptors pyrabactin resistance 1 (PYR1)/PYR1-like/regulatory components of ABA receptors, SNF1-related kinases 2 and Protein phosphatase 2C has been clarified (Ma et al., 2009; Park et al., 2009; Hartung, 2010; Hauser et al., 2011). In addition, ABA also positively regulates heme biosynthesis in *Arabidopsis* (Vanhee and Batoko, 2011; Vanhee et al., 2011). Exogenous ABA addition induces the transient increase of intracellular unbound heme, which is required to activate ABA-8′-hydroxylase, an ABA degradation enzyme to prevent continuous signaling events (Vanhee and Batoko, 2011; Vanhee et al., 2011). At the same time, ABA induces a heme-scavenger protein, tryptophan-rich sensory protein-related protein O (TSPO), to quench excess unbound heme that may cause oxidative damage to the cell (Vanhee and Batoko, 2011; Vanhee et al., 2011).

Abscisic acid has also been found in eukaryotic algae and cyanobacteria. Thus, ABA signaling could be very ancient, but the physiological significance has been poorly understood. In a previous study, we analyzed ABA function in the unicellular red alga *Cyanidioschyzon merolae*, which has available complete nuclear, mitochondrial, and chloroplast genome sequences and molecular genetic tools (Kuroiwa, 1998; Matsuzaki et al., 2004; Nozaki et al., 2007; Kobayashi et al., 2010). In *Arabidopsis* and other land plants, ABA can be synthesized from the common precursor zeaxanthin by three enzymes, zeaxanthin epoxidase (ZEP), 9-*cis*-epoxycarotenoid dioxygenase (NCED), and short-chain dehydrogenase/reductase (SDR). The *C. merolae* genome was found to encode orthologous proteins for NCED and SDR, and the presence of zeaxanthin was previously confirmed in *C. merolae* (Cunningham et al., 2007). Therefore, we checked for endogenous ABA from *C. merolae* culture, and found that *C. merolae* accumulates ABA under the salt stressed condition (Kobayashi et al., 2016). As the NCED knock out strain could not accumulate ABA, the common ABA biosynthetic pathway as in land plants was confirmed in *C. merolae*. While no orthologous gene encoding ZEP was specified from the *C. merolae* genome, it was reported that an *Arabidopsis* plant lacking the functional ZEP still accumulates ABA (Barrero et al., 2005). Thus, there is possibly another type enzyme that directs the reaction, which could be common with the *C. merolae* enzyme.

The addition of exogenous ABA induced a block in the cell-cycle G1/S transition and the homologous *TSPO* gene’s expression. Because TSPO scavenges the intracellular unbound heme in *Arabidopsis*, we wondered whether this unbound heme was required for the cell-cycle initiation, and found that the inhibitory effect by ABA was canceled by the addition of exogenous heme. Thus, ABA prevents the cell-cycle G1/S transition through the reduction of intracellular unbound heme accumulation in *C. merolae* (Kobayashi et al., 2016). As the interconnection among ABA, TSPO, and heme was likely conserved between primitive algae and land plants, the regulatory scheme is likely an ancient trait of ABA signaling conserved during plant evolution. However, the underlying mechanism of ABA-induced heme accumulation has not been clearly elucidated. In this study, we examined the expression of tetrapyrrole biosynthetic genes and the cellular contents of tetrapyrrole intermediates, and hypothesize that ABA affects the tetrapyrrole contents through transcriptional control.

## Materials and Methods

### Materials and Culture Conditions

Cells of *C. merolae* 10D were cultured and their growth synchronized as described previously (Kobayashi et al., 2010).

### Quantitative PCR

Cells were cultured under constant light or synchronizing conditions, with or without ABA (10 μM). Total RNA was extracted from *C. merolae* cells as described previously (Kobayashi et al., 2011). First-strand synthesis of cDNA was performed using 5 μg RNA and random primers with ReverTra Ace (Toyobo, Osaka, Japan), and the abundance of the each transcript was quantified using real-time PCR. Real-time PCR was performed as described previously (Kobayashi et al., 2016), using the primers shown in Supplementary Table S1.

### Measurement of Tetrapyrrole Molecules

Protoporphyrin IX (ProtoIX), magnesium-protoporphyrin IX (Mg-ProtoIX), and chlorophyll-a were measured by high-performance liquid chromatography (HPLC) as described previously (Zapata et al., 2000), with minor modifications. Synchronized cells were homogenized in 80% acetone and centrifuged at 10,000 ×*g* for 5 min. The supernatant was mixed with water to a final concentration of 75% before the HPLC analysis. According to the method of Zapata et al. (2000), pigments were separated on a reversed-phase column, Symmetry C8 (150 mm × 4.6 mm; Waters, Milford, MA, USA) using a Nexera X2 HPLC system (Shimadzu, Kyoto, Japan). Mg-ProtoIX was detected with an excitation wavelength at 417 nm and emission at 600 nm. ProtoIX was detected with an excitation wavelength at 400 nm and emission at 635 nm. Chlorophyll-a was detected by measuring the absorbance at 410 nm. Standard curves were made using authentic standards.

## Northern Blot Analysis

Total RNA preparation and northern blot analyses were performed as described previously (Kobayashi et al., 2011). Gene-specific probes for northern blot analyses were generated with specific primers (Supplementary Table S1) and *C. merolae* genomic DNA as the template.

## Results and Discussion

### Genes Involved in Heme Homeostasis in *C. merolae*

**FIGURE 1:**
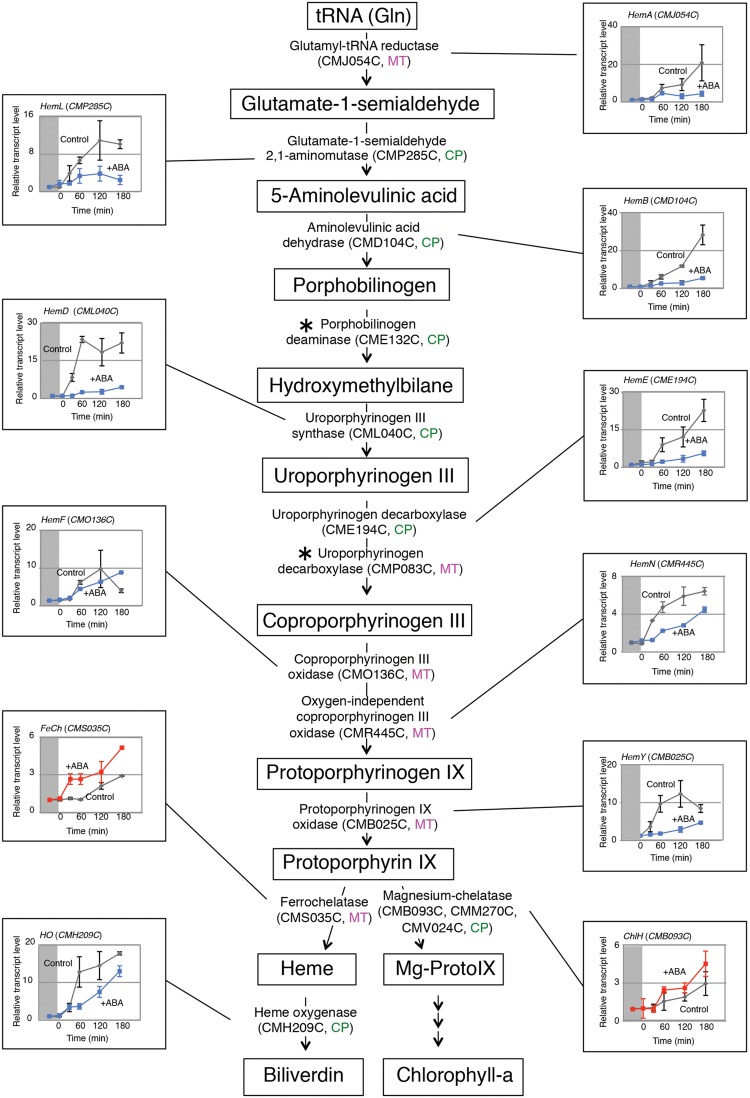
**ABA affected the accumulation of heme-related gene transcripts.** The tetrapyrrole biosynthetic pathway is schematically represented. *C. merolae* cells were dark adapted for 16 h, and the transcript accumulation for each gene was monitored after illumination by quantitative PCR in the absence or presence of ABA. Cells were sampled at the indicated times, and ABA (10 μM) was added 30 min before the light illumination. Data represent the average of three independent experiments (*n* = 3 *SD*). Boxes indicate tetrapyrrole intermediates, and the localization of each enzyme is indicated as in the mitochondrion (MT) or chloroplast (CP) as predicted (Watanabe et al., 2013). The light-dependent increase in the transcript levels were repressed (blue) or activated (red) by ABA addition. Asterisks indicate genes whose transcripts were not detected by the quantitative PCR analysis.

Because most known ABA responses are mediated by the transcriptional regulation of nuclear genes, we examined whether this was also the case for the ABA induced-heme increase. In *C. merolae*, the tetrapyrrole biosynthetic pathway was previously analyzed and localization of each enzyme to specific cell compartments was proposed (Watanabe et al., 2013) (**Figure 1**). Here, we focused on 13 enzymes, glutamyl-tRNA reductase (HemA; CMJ054C), glutamate-1-semialdehyde 2, 1- aminomutase (HemL; CMP285C), aminolevulinic acid dehydrase (HemB; CMD104C), porphobilinogen deaminase (CME132C), uroporphyrinogen III synthase (HemD; CML040C), uroporphyrinogen decarboxylase (HemE; CME194C, CMP083C), coproporphyrinogen III oxidase (HemF; CMO136C), oxygen-independent coproporphyrinogen III oxidase (HemN; CMR445C), protoporphyrinogen IX oxidase (HemY; CMB025C), ferrochelatase (FeCh; CMS035C), heme oxygenase (HO; CMH209C), and, three Mg-chelatases, ChlH (CMB093C), ChlD (CMM270C), and ChlI (CMV024C), that were considered to be involved in the heme homeostasis. These enzymes are encoded by the nuclear genome, except for one of the three Mg-chelatase subunits, ChlI.

### Accumulation of the Heme-Related Gene Transcripts Was Affected by ABA

We first incubated *C. merolae* cells under dark conditions, and the accumulations of heme-related gene transcripts were examined after illumination by quantitative PCR in the absence or the presence of ABA. We could not detect transcripts for two genes, *CME132C* and *CMP083C*, probably because of the limited transcript abundance. For the other genes examined, the detected transcripts increased in response to light while the relative increase and the time course profiles differed for each gene (**Figure 1**). However, the effect of ABA was divided into two categories: ABA repressively affected the light-dependent increase of most gene transcripts, while conversely activating the accumulation of *FeCh* and *ChlH* gene transcripts. As FeCh is the enzyme directly responsible for heme biosynthesis, the activation by ABA may result in the increased heme accumulation. HO is an enzyme involved in heme degradation (Shan et al., 2004; Shekhawat and Verma, 2010), and thus the decrease of *HO* transcripts in the presence of ABA is also consistent with heme accumulation. However, the relevance of the *ChlH* transcript increase, which encodes for a subunit of Mg-chelatase for the chlorophyll biosynthesis branch, is not clear. As we initially only examined the *ChlH* transcripts, we further checked for the expression of other Mg-chelatase subunit genes, *ChlD* and *ChlI*, by northern blot analysis, and confirmed that their transcripts’ accumulation was also activated by ABA (Supplementary Figure S1).

### ProtoIX and Mg-ProtoIX Pools Were also Affected by ABA

**FIGURE 2:**
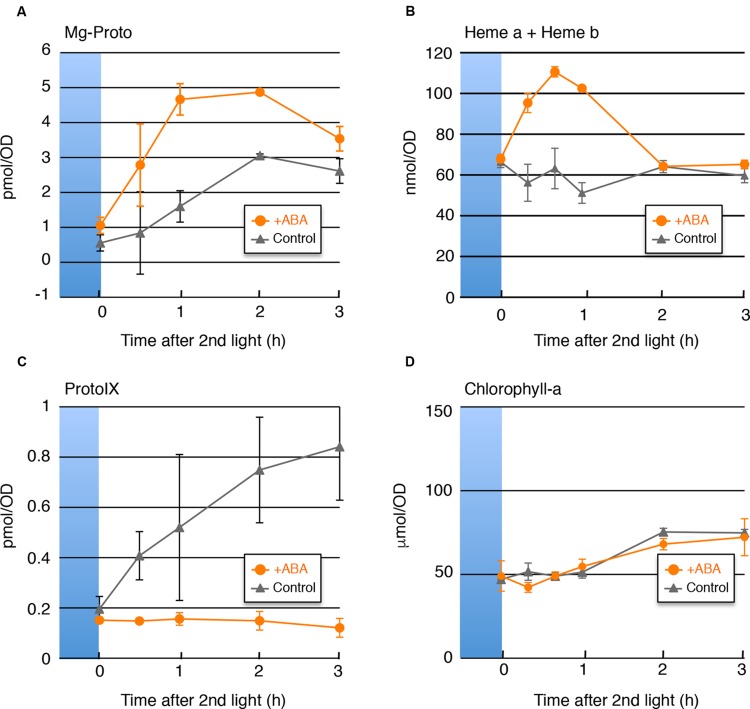
**Cellular Contents of Mg-ProtoIX, Heme, ProtoIX, and Chlorophyll-a.** Changes in accumulation of **(A)** Mg-ProtoIX, **(C)** ProtoIX, and, **(D)** Chlorophyll-a in the absence (gray) or presence (orange) of ABA are shown. The sampled conditions were as in **Figure 1**. **(B)** Changes in heme (heme a and heme b) accumulation (from Kobayashi et al., 2016) are also shown compared with previously obtained results. Each data point represents the average of three independent experiments (*n* = 3 *SD*). The *x*-axis indicates the amounts in 1 mL of culture medium (OD_750_ = 1).

If the transcripts levels explain the change in the heme content, then not only would the increase in heme be expected in the presence of ABA, but also the decrease in ProtoIX and the increase in Mg-ProtoIX. To test this hypothesis, we determined the cellular contents of heme, ProtoIX and Mg-ProtoIX, as well as chlorophyll-a as a control, under the same conditions as in **Figure 1**. As shown in **Figure 2**, the ABA-induced decrease in ProtoIX and increases in heme and Mg-ProtoIX were observed, which was consistent with the gene expression changes. The depletion of ProtoIX, the direct precursor of these compounds, explains the transient nature of the heme and Mg-ProtoIX increases. The chlorophyll-a content was not affected by ABA probably because the cellular content was rather abundant as compared with other tetrapyrrole intermediates and not easily affected by the change in the biosynthesis rate.

### TSPO May Scavenge the ABA-Induced Unbound Mg-ProtoIX

In a previous study, we showed that a transient increase in ProtoIX or Mg-ProtoIX induces nuclear DNA replication (Kobayashi et al., 2009). This could have indicated that ABA induces nuclear DNA replication, but this was not the case, and ABA inhibited cell-cycle initiation (Kobayashi et al., 2016). The total heme content was increased by ABA, but the unbound heme that is required for cell-cycle initiation was decreased through the induction of the heme scavenger protein TSPO (Kobayashi et al., 2016). TSPO has an affinity not only to heme but also for dicarboxylic porphyrins, including Mg-ProtoIX (Papadopoulos et al., 2006; Vanhee and Batoko, 2011). Thus, even when the total Mg-ProtoIX content increased, its unbound form, required for the signaling events, was likely quenched by TSPO and not available for cell-cycle activation.

### Physiological Significance of the Heme Accumulation

It was previously shown that salt stress induced ABA accumulation in *C. merolae* (Kobayashi et al., 2016). While ABA is the phytohormone that transmits the stress signal to other cells in land plants, ABA in *C. merolae* is likely an intracellular signaling molecule since ABA rapidly loses its activity in the sulfur acidic culture medium (Kobayashi et al., 2016). As the signaling molecule, it is important to prevent the continuous ABA signaling, and thus the degradation enzyme ABA-8′-hydroxylase should be activated subsequently. In addition, salt stress induces generation of oxygen radicals, which needs to be scavenged by enzymes such as catalase and peroxidase. These enzymes require heme as the prosthetic group, and therefore it is reasonable that salt stress-induced ABA activates the heme accumulation. The excess unbound heme itself may cause the radical formation, which requires quenching by the ABA-induced TSPO.

### Evolutionary Implications

The interrelationship among ABA, heme, and TSPO was first shown in *Arabidopsis* (Vanhee and Batoko, 2011; Vanhee et al., 2011), and a similar scheme was subsequently revealed in *C. merolae* (Kobayashi et al., 2016). Thus, this regulatory scheme was likely conserved during plant evolution. Presently, two issues remain to be addressed from mechanical and evolutionary points of view. First, although heme activates the cell-cycle G1/S progression in *C. merolae*, the presence of a similar mechanism has not been found in land plants. Future studies on the underlying molecular mechanism in *C. merolae* would clarify the significance for plant cell-cycle control. Second, an increase in the heme content was observed in *C. merolae*, as well as in *Arabidopsis*, but almost no information on the underlying mechanism in *Arabidopsis* has been provided. ABA-induced changes in the *Arabidopsis* transcriptome have been reported previously (Matsui et al., 2008), but it is difficult to determine the relevance to the heme content because the experimental time scale was rather different. Whether the expression of tetrapyrrole biosynthetic genes is regulated as in *C. merolae* needs to be clarified. The elucidation and comparison of these whole schemes in *C. merolae* and *Arabidopsis* would provide insights on the evolution of plant cell physiology.

## Author Contributions

YK performed the experiments and contributed to the writing of the manuscript. KT designed and had overall responsibility for the study, and wrote the manuscript.

## Conflict of Interest Statement

The authors declare that the research was conducted in the absence of any commercial or financial relationships that could be construed as a potential conflict of interest.
