# Supplementary material for: Transcriptional Regulation of Tetrapyrrole Biosynthetic Genes Explains Abscisic Acid-Induced Heme Accumulation in the Unicellular Red Alga Cyanidioschyzon merolae
Source: Front Plant Sci. 2016 Aug 29;7:1300. doi: 10.3389/fpls.2016.01300 (PMC5002421; doi:10.3389/fpls.2016.01300)
Supplement: Supplementary file 1 [file Table_1.DOCX]

**Supplementary table**

**Table S1. Primer list**

| For quantitative PCR | Gene | Primer name | Sequence |
| --- | --- | --- | --- |
|  | *HemA* (*CMJ054C*) | HemA J054-q-F | aaaatctgttgcgggatgag |
|  |  | HemA J054-q-R | cgctcgtcgatgtcttgtaa |
|  | *HemL* (*CMP285C*) | HemL P285-q-F | acaggaaagacccatgttcg |
|  |  | HemL P285-q-R | gccgtccacgtcaactatct |
|  | *HemB* (*CMD104C*) | HemB D104-q-F | acatcgctctggacccatac |
|  |  | HemB D104-q-R | agcgtaggaaacgatcgaga |
|  | *HemC* (*CME132C*) | HemC E132-q-F | agaaccccgctattgttgtg |
|  |  | HemC E132-q-R | atatccggaggcaggaaagt |
|  | *HemD* (*CML040C*) | HemD L040-q-F | aggttcggacagatgcagac |
|  |  | HemD L040-q-R | ggcaacttctcaacgggtaa |
|  | *HemE* (*CME194C*) | HemE E194-q-F | acatgaacgcataccgtgaa |
|  |  | HemE E194-q-R | aaaggcgtcgacttgtatgg |
|  | *HemE2* (*CMP083C*) | HemE2 P083-q-F | acgtggaagaattggtacgc |
|  |  | HemE2 P083-q-R | gcaaacacaacgatcaatgg |
|  | *HemF* (*CMO136C*) | HemF O136-q-F | ggcgtgtttttcgatgattt |
|  |  | HemF O136-q-R | cccttctgctggttgatgat |
|  | *HemN* (*CMR445C*) | HemN R445-q-F | ctcgtagcggacacactgaa |
|  |  | HemN R445-q-R | ccaggttccaatccttgcta |
|  | *HemY* (*CMB025C*) | HemY B025-q-F | ccagatggcgaacgagttat |
|  |  | HemY B025-q-R | aactcgacgaccagaccatc |
|  | *FeCh* (*CMS035C*) | HemH S035-q-F | ccgctgttgcactattctca |
|  |  | HemH S035-q-R | gtaggcgggatggtcataga |
|  | *ChlH* (*CMB093C*) | Chl B093-q-F | cccagcaccaagagaaacat |
|  |  | Chl B093-q-R | tcatccagcagacagacgac |
|  |  |  |  |
| For northern blot analysis | *ChlD* (*CMM270C*) | M270-probe-F | accctcgtgaatttgtacgc |
|  |  | M270-probe-R | cgctcgtcggaaaatatgat |
|  | *ChlI* (*CMV024C*) | V024-probe-F | gggagatcgtggtacaggaa |
|  |  | V024-probe-R | ggatcacggtcaaaagcact |
