# Supplementary material for: Transcriptional Regulation of Tetrapyrrole Biosynthetic Genes Explains Abscisic Acid-Induced Heme Accumulation in the Unicellular Red Alga Cyanidioschyzon merolae
Source: Front Plant Sci. 2016 Aug 29;7:1300. doi: 10.3389/fpls.2016.01300 (PMC5002421; doi:10.3389/fpls.2016.01300)
Supplement: Supplementary file 2 [file Image_1.PDF]

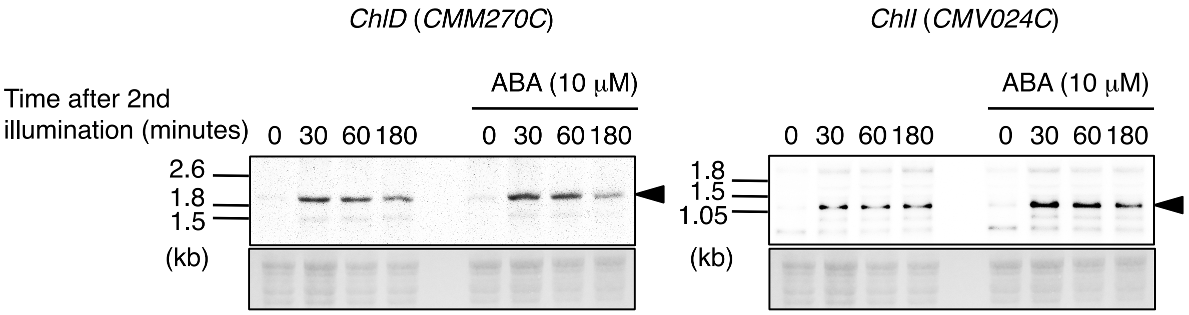

Supplementary figure S1. Changes in the accumulations of *ChlD* and *ChlI* transcripts in response to ABA

Northern analysis of *ChlD* and *ChlI.* Synchronized cells were sampled at the indicated times. The lower panel shows the methylene blue-stained total RNA. Each lane contains 5 μg of total RNA. Arrowheads indicate the predicted sizes of the transcripts.
